# Editorial: Advances in Genomics of Crossbred Farm Animals

**DOI:** 10.3389/fgene.2021.709483

**Published:** 2021-08-05

**Authors:** Xiao-Lin Wu, Shuhong Zhao

**Affiliations:** ^1^Council on Dairy Cattle Breeding, Bowie, MD, United States; ^2^Department of Animal and Dairy Sciences, University of Wisconsin, Madison, WI, United States; ^3^College of Animal Science and Technology, Huazhong Agricultural University, Wuhan, China

**Keywords:** crossbreeding, genomics, GWAS, genomic prediction, livestock, SNP

## Introduction

Crossbreeding is a common strategy to promote animal production (Sheridan, 1981). In the past century, crossbreeding has been commonly conducted to produce commercial pigs and poultry as human food. In subtropical countries, crossbred cattle have been developed that combine the production performance of Taurine cattle with the tropical adaption of Zebu cattle. Composite cattle are developed by crossing two or more purebred breeds, aiming at exploiting breed complementarity and retaining some heterosis (hybrid vigor) in future generations. Dairy cattle are mostly purebred, but crossbred dairy cattle are becoming increasingly popular in recent years (VanRaden et al., 2020; Khansefid et al.). Strategically, crossbreeding is a potential approach to improve sustainability in animal breeding by reducing inbreeding and enhancing fertility, survival, and other functional traits (Sørensen et al., 2008).

Genomics is an interdisciplinary field of biology focusing on the studies of genomes (Culver and Labow, 2002). A genome is an organism's complete set of DNA, including all of its genes. Unlike classic genetics, which focuses on individual genes and their roles in inheritance, genomics deals with the collective characterization and quantification of all of an organism's genes, their interrelations, and their influence on the organism. From the genomics perspective, crossbred animals differ considerably from purebred animals because their genome is a mosaic of genome regions inherited from their purebred ancestors. Thus, genomics solutions for crossbred animals need to be different. For example, ancestry estimation or genomic breed composition (GBC) in purebred animals is primarily motivated for breed registries and the identification of purebred animals when the pedigree is missing or incomplete. In contrast, the estimated GBC for crossbred animals are used to infer their genomic make-ups from their ancestors. Such information can help estimate heterozygosity, understand their breeding history, and make management decisions for crossbreeding programs. The breeding objective with purebred animals is to increase additive genetic gains, but non-addtive genetic effects such as dominance and epistasis effects are pivotal to produce crossbred animals of high-performance market values.

The past decades have witnessed many milestone discoveries in animal genomics which have fundamentally revolutionized many aspects of animal breeding and production (Rexroad et al., 2019). Nevertheless, there are far more questions still unanswered. This Research Topic represented an effort toward enhancing the understanding and applications of crossbred genomics. It included 25 papers, covering several aspects of the crossbred genomics in farm animals.

## Interpretations of Genomic Breed Composition and “The Impure Purebred Paradox”

Genomic breed composition (GBC) of an individual animal refers to the partition of its genome according to the inheritance from its ancestors or ancestral breeds. The U.S. Council on Dairy Cattle Breeding (CDCB) uses an alternative term, namely Breed Base Representation (BBR), which is the adjusted genomic breed composition on each of five dairy breeds (Ayrshire, Brown Swiss, Guernsey, Holstein, and Jersey) as the potential parents (VanRaden and Cooper, 2015). Interpretations of GBC depend on the estimation methods. For example, admixture model postulates that an observed genotype for a progeny is an instance of a multinomial distribution, with genotype probability being a mixture governed by allelic frequencies of the ancestors. Hence, GBC are estimated by the weights or admixture coefficients (Bansal and Libiger, 2015). Linear regression estimated GBC of to be adjusted regression coefficients of coded genotypes for a progeny on the ancestral allele frequencies, and bounded between 0 and 1 (Chiang et al., 2010; Kuehn et al., 2014). A genomic prediction model estimates the SNP effects on candidate ancestry breeds as binary or categorical traits. GBC equals to the total genomic value for an animal pertaining to each ancestry breed (Akanno et al., 2017; Li et al., 2020). Wu et al. proposed a causal interpretation of GBC based on path theory, which decomposed the relationships between ancestors and their progenies into direct and indirect breed (path) effects. GBC was measured by relative ratio of direct (D-GBC) and combined (C-GBC) breed determination, respectively, from each putative ancestry breed to a progeny. C-GBC included direct breed effects and indirect breed effects due to genomic similarities. The estimated D-GBC and C-GBC were comparable when the ancestry breeds had a very distant relationship, and they corresponded well to the estimated GBC from linear regression and admixture model. However, large differences arose between D-GBC and C-GBC when ancestors were highly correlated. Overall, the estimated C-GBC was closer to the estimated GBC from linear regression and admixture models than D-GBC.

In reality, all the modern cattle breeds are correlated because they share common ancestors. The same is true with other farm animal species. The estimated GBC for a purebred animal is not always 100%. This phenomenon was referred to as “the Impure purebred Paradox” (Wang et al.). In the U.S. dairy genetic evaluation, for example, the reference population for a dairy breed consisted of animals with a BBR no ≥ 94% for that breed, and animals with BBR no ≥ 90% received single-breed genomic evaluation (Wiggans, 2021). This was because the current methods tend to produce a small GBC value to a non-ancestry reference breed. The more significant the genomic similarity, the more noise. Statistically, this situation was an indication of increased false-negative error rates in the identification of purebred animals. Wang et al. (2020) proposed applying regularization in admixture models to estimate GBC for purebred animals. Regularized admixture methods produced sparse solutions of admixture coefficients, thus effectively imposing penalties on small, non-essential components due to genomic similarity. The non-convex penalty outperformed the L1 norm penalty to suppress the noise in the estimated GBC.

Several issues are not addressed adequately. Firstly, accurately assessing GBC requires knowing or reliably estimating the allelic frequencies for the base population when the ancestor breeds were developed, because they are not observable. Secondly, while high-density SNP genotypes were used to estimate GBC, the impact of SNPs in high linkage disequilibrium on the estimated GBC has not been well-documented. In admixture models, for example, the likelihood is computed assuming mutual independence of SNP loci, but this assumption does not hold with high-density SNP arrays. Finally, the current methods do not estimate GBC exactly based on genomic similarities identical-by-descend (IDB) between a progeny and the ancestry (reference) breeds. Rather, they reflect more of genomic similarities identical-in-statue (IIS).

## Limited Efforts With Differential Gene Expression Profiling

Expression profiling is a logical next step after genome sequencing, which reveals the activity of genes in hundreds and thousands and depicts a global picture of cellular functions (Subramanian et al., 2005). Expression profiling experiments involve measuring relative mRNA abundance in two or more experimental conditions. Altered gene expression suggests a change for the protein coded by the mRNA, probably indicating a homeostatic response or a pathological condition.

There were only three papers addressing differential gene expression in this Research Topic. Chen et al. compared the microRNA (miRNA) profiles of pectoral muscle in chickens at pre- to post-natal stages. Cui et al. identified differentially expressed miRNAs between cattle with high vs. low milk protein and fat percentages. A miRNA is a small single-stranded non-coding RNA molecule containing about 22 nucleotides in animals. It functions in RNA silencing and post-transcriptional regulation of gene expression. First discovered in the early 1990s (Lee et al., 1993), miRNAs were not recognized as a distinct class of biological regulators until the early 2000s (Bartel, 2004). Chen et al. investigated the expression pattern of pituitary-derived circular RNAs and their functions in Landrace × Yorkshire crossbred pigs. A circular RNA is a single-stranded RNA that forms a covalently closed continuous loop. Some circular RNAs have shown potential as gene regulators.

The size and complexity of these gene expression experiments are crucial to reach reliable interpretations. In reality, however, lacking sufficient sample sizes was mainly related to financial constraints, which led to reduced statistical power of the experiment and difficulty to identify essential but subtle changes, and limited the extent to which experiments performed in different laboratories appeared to agree. Different gene expression between purebred and crossbred animals may have implications on the expression of heterosis, but relevant studies are missing in this Research Topic.

## The Journey Continues With Dissecting Quantitative Trait Variation and Genetic Architecture

Quantitative trait locus (QTL) mapping aims at characterizing chromosomal regions or genes responsible for quantitative traits and diseases in terms of genomic positions, effects, and numbers. A simple QTL mapping experiment starts with crossing two parental lines differing in their trait values and marker variants. Segregated QTLs are observed and mapping in the consequent backcrosses or F2 population. Improved strategies, such as advanced intercross lines (AIL) (Darvasi and Soller, 1995), can increase the precision of quantitative trait loci (QTL) mapping due to more recombination events. An AIL is created by successive generations of pseudo-random mating after the F2 generation, and recombination events are accumulated continuously between generations. Wang et al. evaluated a nine-generation AIL derived from two divergent outbred chicken lines. Their results showed that the founder genomes were sufficiently shuffled in the F9 generation. This AIL reference population yielded a considerably narrower for mapped QTL than the F2 generations.

Genome-wide association studies (GWAS) emerged as a powerful tool to investigate associations between quantitative traits (including diseases) and genetic markers on the entire genome (Ozaki et al., 2002; Klein et al., 2005). There were six GWAS studies in this Research Topic, covering cattle, pigs, and chickens, respectively. Rezende et al. identified five genomic regions associated with carcass and meat quality traits in a crossbred Angus-Brahman population. Gao et al. found significant loci for meat quality traits in pigs. Carcass and meat quality are important traits that drive profitability and consumer demand for beef and pork. They are expensive to measure and unavailable until late in life or after the animal was harvested. Hence, genetic improvement of carcass and meat quality traits is not viable through traditional phenotypic selection, but these traits are perfect candidates for marker-assisted selection or genomic selection. Instead of alive measurement of carcass and meat quality traits, Grigoletto et al. attempted to localize chromosomal regions associated with non-invasive, ultrasound-based carcass and meat quality traits in Montana Tropical Composite beef cattle. Li et al. identified several candidate genes that are associated with metabolites, which are intermediate or end product (usually small molecules) of metabolism, in crossbred beef cattle. Li et al. found significant loci in chromosome 1 and chromosome 4, which explained 6.36 and 4.25% of the phenotypic variance of birth weight. Nie et al. revealed seven significant SNPs spanning a ~0.29 Mb, harboring 14 candidate genes for tail feather color, in dwarf chickens.

Selection tends to cause specific changes in the patterns of variation among selected loci and in neutral loci linked to them, leaving genomic footprints known as selection signatures (Kreitman, 2000). Such information helps understand how genomes were shaped during the breeding history and localize functional genes/genomic regions. Singh et al. identified eleven common regions harboring genes associated with production and adaptation in an Indian composite cattle breed developed by crossbreeding taurine dairy breeds with native indicine cattle. Their results suggested more substantial selective pressure on regions responsible for adaptation compared to milk yield. Paim et al. estimated the genomic composition of the regions identified as selected (selective sweeps) using a chromosome painting approach. Selected genomic regions as selection signatures for founder breeds were identified as well. van der Nest et al. identified ten candidate regions potentially under strong positive selection, harboring genes for health and production, in South African Simbra cattle (5/8 Taurine and 3/8 Indicine). Ganteil et al. assessed the patterns of runs of homozygosity (ROH) in animals from three-way crossbreeding. ROH are continuous stretches of homozygous genotypes in a diploid genome, and their quantification reflects autozygosity, which occurred when two parents shared at least one common ancestor (Peripolli et al., 2017).

Given the polygenic nature of quantitative traits and disease, an adequate sample size for GWAS often tends to be very large (Nishino et al., 2018). In reality, however, assembling large sample sizes is not always possible, particularly for carcass and meat quality traits because they are difficult or expensive to measure. A similar challenge arises when conducting GWAS in isolated small populations. Hence, literature synthetic or meta-analytical methods provides an alternative to incorporate data from multiple studies and arrive at more reliable conclusions by utilizing publicly accessible databases (Wu and Hu, 2012). Population stratification is another concern with GWAS, which often result in spurious associations if not properly accounted for. Population stratification can happen in large GWAS when perfect matching of cases and controls is virtually impossible. It is also likely to occur when studying recently admixed populations and variants with very small effect sizes.

GWAS do not necessarily pinpoint causal variant and genes, because most association signals map to non-coding regions of the genome (Hindorff et al., 2009; Mahajan et al., 2018). Functional characterization of genetic variants is needed to move from statistical association to causal variants and genes, especially in the non-coding genome. Computational methods are used to predict the regulatory effect of non-coding variants on the basis of functional annotations. Target genes can be identified using chromatin immunoprecipitation and chromosome conformation capture methods, and experimentally validated using cell-based systems and model organisms. A development in the past decade combined QTL analyses with gene expression profiling, i.e., by DNA microarrays. Such expression QTLs (eQTLs) describe cis- and trans-controlling elements for the expression of often disease-associated genes (Westra et al., 2013).

Most GWAS have been conducted using SNP arrays because they are cost-effective. Nevertheless, whole genome sequencing (WGS) permits studying the full frequency spectrum of variants, including rare variants that are difficult to capture by SNP arrays. We anticipate that, as the cost of WGS continues to decline, GWAS using WGS will eventually replace GWAS using SNP arrays. Until then, the majority of the common variants and a substantial fraction of the low-frequency and rare variants that contribute to disease risk can be identified using affordable SNP arrays combined with imputation to increasingly large WGS reference panels (Tam et al., 2019). Low-pass sequencing (i.e., an average depth <1 × coverage) combined with genotype imputation have been proposed as an alternative to genotyping arrays which showed increased power for GWAS (Pasaniuc et al., 2012; Gilly et al., 2019).

## Expending the Horizons of Genomic Prediction For Crossbred Animals

Quantitative traits are determined by thousands of genes with small effects, which are often difficult to detect (Manolio et al., 2009; Slatkin, 2009). The merge of genomic selection led to a revolutionary paradigm shift in animal breeding (Meuwissen et al., 2001, 2016). With a sufficient number of markers covering the whole genome, genomic selection concentrates on estimating their total effect rather than testing single loci for their significance. Most genomic evaluations, say for dairy cattle, are separate by breed and crossbreds usually are not included except for the multibreed evaluation in New Zealand (Winkelman et al., 2015). Crossbred animals were removed based on counts of breed check markers (Wiggans et al., 2010). On the other hand, there has been an increasing interest in genomic predictions for crossbred animals in recent years (Sørensen et al., 2008). Starting from April 2019, CDCB offered a genomic evaluation for crossbred dairy cattle on more than 50 traits yet limited to crosses of five dairy breeds (VanRaden et al., 2020). Crossbred evaluations were averages of direct genomic values computed using marker effects for each of the five pure breeds, weighted by the animal's genomic breed composition (VanRaden et al., 2020).

Purebred prediction models do not fully meet the need for evaluating crossbred animals because they are limited to additive genetic effects only. On the other hand, non-additive genetic effects such as dominance and epistasis effects are essential components contributing to the crossbred performance. Stock et al. gave a literature review of genomic models for analyzing livestock crossbred data. Genomic models for crossbred animals extend purebred models with more complexity, such as the inclusion of dominance effects, breed-specific effects, imprinting effects, and the joint evaluation of purebred and crossbred performance data. A two-way cross additive model is the simplest example (Christensen et al., 2014), where the additive genetic value of a crossbreed animal, captured by SNP effects, is decomposed into a contribution that comes from the sire (or sire line) and a contribution from the dam (or dam line), plus a Mendelian sampling term. This basic model can be extended to three-way (Christensen et al., 2019) and four-way crossings and include dominance effects as well. SNP effects are assumed to be either the same or different SNP effects across pure lines. The latter are referred to as BOA (breed-of-origin of alleles) models (Sevillano et al., 2016, Lopes et al., 2017). Including dominance effects is in general advisable, leading to higher accuracy (e.g., Zeng et al., 2013; Xiang et al., 2016). Nevertheless, available studies are not sufficiently conclusive as to which existing method is most suitable for a specific crossbreeding or a genetic trait architecture. Deep learning methods are non-linear models providing flexibility to adapt to complicated relationships between data and output (reviewed by Montesinos-López et al., 2021). They are particularly appealing for crossbred predictions, but not covered in this Research Topic.

Apart from statistical models, the establishment of an appropriate reference population is also crucial to crossbred predictions. In dairy cattle, for example, genotype data are huge and unbalanced between breeds. Dairy genomic evaluations are conducted several times a year. Hence, combing genotypes from multiple breeds imposes great computational challenge. Besides that, multiple-breed predictions are less accurate than within-breed predictions. Training on crossbred animals can increase the prediction accuracies for crossbred animals (Esfandyari et al., 2015), but collecting data from crossbred animals is often difficult and expensive. Optimal training strategies for crossbred predictions remain to be exploited. Alvarenga et al. showed that including purebred and crossbred animals in a joint training population yielded the higher accuracies and lower biases than only training on purebred animals in single-trait or multiple-trait analyses. The multiple-trait model treated purebred and crossbred phenotypes as different traits. Khansefid et al. proposed a strategy by equalizing breed contributions in a mixed dairy breed reference of Holsteins, Jerseys, and their crossbreds, instead of a Holstein-dominated reference. Their results showed improved genomic predictions for crossbred and purebred animals using this strategy. With a support vector machine (SVM) regression model, Tusell et al. also showed increased accuracies by including crossbred information for training when predict the performance of purebred and crossbred pigs. As the genomic data are accumulating indefinitely, the computational challenge will extremely high. Hence, optimal sample selection is worth exploiting, which aims at choosing subsets of training samples that give the same or comparable prediction accuracy as the whole training set. This concept was proposed by Frankel (1984) to select a subset of the data that is representative of the whole resource by removing redundant or highly correlated samples. Also, high-performance computing offers a solution to bypass the computational bottleneck (Wu et al., 2011, 2012; Coninck et al., 2014).

Single-step genomic BLUP enables the inclusion of marker genotypes into the well-established BLUP methods, which often leads to increased prediction accuracies (Legarra et al., 2009; Misztal et al., 2009). This method has been challenged by defining the genetic base when pedigree and genomic information are used simultaneously. For predicting crossbred performance, the challenge becomes how to quantify relationships between different lines compositions and appropriately define different base generations. One solution is to use metafounders, which are pseudo-individuals, that describe the genetic relationship between the base population individuals (Christensen, 2012; Legarra et al., 2015). Junqueira et al. showed that using metafounders increased the accuracy of GEBV and the rate of genetic gain for tick resistance using single-step genomic BLUP in multi-breed beef cattle populations. They defined four metafounders, each for the three pure breeds (Hereford, Bradford, and Zebu) and the fourth metafounder assigned to the remaining base animals with an unknown breed of origin.

Genomic selection in indigenous or minor breeds is often limited by the number of animals with genotypes and phenotypes for training. Combining animals from breeds with similar backgrounds or development history can increase the training population sizes and prediction accuracy. Oliveira et al. reported a moderate genetic connectedness between Norwegian White Sheep and New Zealand Composite Sheep with similar development history, based on the consistency of gametic phase and other genetic diversity metrics. Their results suggested a promising opportunity for cross-country genomic selections. Gebrehiwot et al. found moderate to high genomic composition of European Bos taurus cattle in Western African crossbred cattle. Hence, the genomic information from European Bos taurus cattle can be borrowed to improve genotype imputation and genomic selection in the Western African crossbred cattle. While genomics studies are heavily directed toward major livestock species and breeds, genomics tools for minor livestock species and breeds are in need (Das et al.; Gebrehiwot et al.; Yang et al.).

## Conclusions and Prospects

Genomics focuses on the structure, function, evolution, mapping, and editing of genomes (Culver and Labow, 2002). Genomics studies also included studies of intragenomic phenomena such as epistasis, pleiotropy, heterosis, and other interactions between loci and alleles within the genome (Pevsner, 2009). Given such a broad spectrum of genomics domains, the coverage of this Research Topic is very limited. The 25 articles are mostly in the domains of functional genomics and predictive genomics in crossbred animals. The former used available genomic data to describe gene functions and interactions, whereas the latter attempts to predict the performance of individual animals based on low- to high-density genotype data. Studies in structural genomics, epigenomics, and metagenomics in crossbred livestock animals are essential, but they are not addressed in this collection.

Advances in genomics have triggered a revolution in discovery-based research and systems biology concerning complex biological systems. Driving genomics to practice, genomic prediction is at the core of enhancing animal breeding and farming management. We anticipate more efforts to specifically exploit genomic prediction models and cost-effective training strategies for crossbred animals. Innovative genomic mating and crossbreeding is appealing for improving commercial crossbreeding. The objective for crossbreeding is to find optimal combinations which maximize the general combining ability (GCA) from the contributing parental lines and the special combining ability (SCA) between them, penalized by standard deviation of gamete breeding values passed from the parents to the offspring. This type of innovative mating or crossbreeding schemes is expected to produce high-performance and less-variable crossbred animals.

Finally, predicting heterosis remains a topic of interest. Heterosis is an old concept proposed by George Harrison Shull, American botanist and geneticist known as the father of hybrid corn, in 1914 (Shull, 1948). In animal breeding, it refers to crossbred performance superiority relative to the parental average (Lush, 1945). Two competing but not mutually exclusive hypotheses, dominance hypothesis and dominance hypothesis, have been proposed to explain heterosis or hybrid vigor (Crow, 1948). Epigenetic components of hybrid vigor were established recently, pinpointing the involvement of small RNAs in the growth, vigor and adaptation of hybrids (Ni et al., 2009; Baranwal et al., 2012). Heterosis is linearly related to heterozygosity, considered to be 100% in the first generation cross (F1) between two diverse parental breeds. In the following generations, it is measured as retained heterosis or heterozygosity relative to F1 (Dickerson, 1973). Genomic-estimated retained heterozygosity or heterosis (GRH) can be used to match parents to obtain optimized heterosis and produce progeny with improved performance and replacement females with better lifetime productivity (Akanno et al., 2017). A silent feature is that GRH provides an additional metric for the existing purebred genomic evaluation systems, for example, in beef cattle to include crossbred predictions without any infrastructural change (Basarab et al., 2018).

## Author Contributions

X-LW drafted the manuscript, in discussion with SZ. All authors have proof-read the final version.

## Conflict of Interest

The authors declare that the research was conducted in the absence of any commercial or financial relationships that could be construed as a potential conflict of interest.

## Publisher's Note

All claims expressed in this article are solely those of the authors and do not necessarily represent those of their affiliated organizations, or those of the publisher, the editors and the reviewers. Any product that may be evaluated in this article, or claim that may be made by its manufacturer, is not guaranteed or endorsed by the publisher.
